# Enemy of My Enemy: A Novel Insect-Specific Flavivirus Offers a Promising Platform for a Zika Virus Vaccine

**DOI:** 10.3390/vaccines9101142

**Published:** 2021-10-07

**Authors:** Danielle L. Porier, Sarah N. Wilson, Dawn I. Auguste, Andrew Leber, Sheryl Coutermarsh-Ott, Irving C. Allen, Clayton C. Caswell, James A. Budnick, Josep Bassaganya-Riera, Raquel Hontecillas, James Weger-Lucarelli, Scott C. Weaver, Albert J. Auguste

**Affiliations:** 1 Department of Entomology, Fralin Life Science Institute, Virginia Tech, Blacksburg, VA 24061, USA; danip@vt.edu (D.L.P.); swilson3@vt.edu (S.N.W.); dauguste@vt.edu (D.I.A.); 2 Nutritional Immunology and Molecular Medicine Laboratory Institute, Blacksburg, VA 24060, USA; andrew@nimml.org (A.L.); jbassaga@nimml.org (J.B.-R.); rmagarzo@nimml.org (R.H.); 3 Department of Biomedical Sciences and Pathobiology, Virginia-Maryland College of Veterinary Medicine, Blacksburg, VA 24060, USA; slc2003@vt.edu (S.C.-O.); icallen@vt.edu (I.C.A.); caswellc@vt.edu (C.C.C.); jab5771@vt.edu (J.A.B.); weger@vt.edu (J.W.-L.); 4 Center for Emerging, Zoonotic, and Arthropod-Borne Pathogens, Virginia Tech, Blacksburg, VA 24061, USA; 5 Center for One Health Research, Virginia-Maryland College of Veterinary Medicine, Blacksburg, VA 24060, USA; 6 World Reference Center for Emerging Viruses and Arboviruses, University of Texas Medical Branch, Galveston, TX 77555, USA; sweaver@utmb.edu; 7 Institute for Human Infections and Immunity, University of Texas Medical Branch, Galveston, TX 77555, USA; 8 Department of Microbiology and Immunology, University of Texas Medical Branch, Galveston, TX 77555, USA

**Keywords:** flavivirus, insect-specific virus, vaccine, Zika virus, correlates of protection

## Abstract

Vaccination remains critical for viral disease outbreak prevention and control, but conventional vaccine development typically involves trade-offs between safety and immunogenicity. We used a recently discovered insect-specific flavivirus as a vector in order to develop an exceptionally safe, flavivirus vaccine candidate with single-dose efficacy. To evaluate the safety and efficacy of this platform, we created a chimeric Zika virus (ZIKV) vaccine candidate, designated Aripo/Zika virus (ARPV/ZIKV). ZIKV has caused immense economic and public health impacts throughout the Americas and remains a significant public health threat. ARPV/ZIKV vaccination showed exceptional safety due to ARPV/ZIKV’s inherent vertebrate host-restriction. ARPV/ZIKV showed no evidence of replication or translation in vitro and showed no hematological, histological or pathogenic effects in vivo. A single-dose immunization with ARPV/ZIKV induced rapid and robust neutralizing antibody and cellular responses, which offered complete protection against ZIKV-induced morbidity, mortality and in utero transmission in immune-competent and -compromised murine models. Splenocytes derived from vaccinated mice demonstrated significant CD4^+^ and CD8^+^ responses and significant cytokine production post-antigen exposure. Altogether, our results further support that chimeric insect-specific flaviviruses are a promising strategy to restrict flavivirus emergence via vaccine development.

## 1. Introduction

Members of the genus *Flavivirus* (family, *Flaviviridae*) have a near-global distribution and remain among the most important arthropod-borne viruses (arboviruses) affecting human and animal health. Since 2007, the Zika virus (ZIKV) emerged as a serious public health threat in Southeast Asia, the South Pacific and South and Central America [1]. ZIKV infection typically results in inapparent or febrile illness with similar clinical presentation to dengue, but can manifest in more severe forms than febrile illness. ZIKV infection is associated with Guillain-Barré syndrome (a serious and sometimes fatal paralytic disease of the peripheral nervous system [2]) and congenital Zika syndrome (CZS), which includes microcephaly—a major neonatal malformation that is 20 times more likely to occur post-ZIKV infection of pregnant mothers [3]. CZS may also manifest as chorioretinal atrophy or congenital muscular contractures that restrict body movement [4]. In 2016 alone, an estimated 29% of babies born to ZIKV-infected mothers in some regions of Brazil exhibited developmental abnormalities [5,6]. The threat of ZIKV is further exacerbated by its capacity to be transmitted via alternative routes, such as sexual and perinatal, and, possibly, via blood-transfusion [7].

Despite the significant public health impact of flaviviruses, safe and effective licensed vaccines for flaviviruses continue to remain limited, with the exception of live-attenuated vaccines for Japanese encephalitis (JE), dengue and yellow fever, and inactivated vaccines for JE, tick-borne encephalitis and Kyasanur Forest disease (reviewed in [8,9,10]). Several vaccine candidates for ZIKV have been described [11,12,13,14,15,16,17] and a DNA-based vaccine [18,19], a purified-inactivated vaccine (ZPIV) [20,21,22] and a live-attenuated vaccine (rZIKV/D4Δ30-713) (https://clinicaltrials.gov/ct2/show/results/NCT03611946, 2 August 2018) are presently in clinical trials, but none is yet FDA-approved. Each of these vaccines present specific advantages and challenges, but there is a critical need for additional platforms that more delicately balance safety and efficacy, to effectively combat the considerable morbidity associated with ZIKV infection and ultimately reduce its re-emergence potential.

Insect-specific viruses are a taxonomically diverse group of viruses and an underutilized resource in biomedical research. Insect-specific flaviviruses (ISFV) have been studied in the context of superinfection exclusion (i.e., the ability of ISFVs to prevent cells from becoming subsequently infected by another flavivirus) to aid in reducing vector competence of their pathogenic counterparts [23,24], as well as for diagnostic and vaccine development [25,26,27]. The first insect-specific virus used for vaccine and diagnostic development was the alphavirus (*Togaviridae*: *Alphavirus*) Eilat virus [28], which was used to develop a chimera expressing the structural proteins of other alphaviruses such as chikungunya and Venezuelan equine encephalitis viruses [29,30,31]. These chimeras retain the vertebrate replication-incompetent phenotype, provide single-dose immunogenicity and efficacy in murine and nonhuman primate models and serve as safe and effective antigens for diagnostics.

To address the need for additional flavivirus vaccine strategies, we developed a new ISFV-vectored platform based on a recently discovered ISFV, Aripo virus (ARPV) [32]. Herein, we describe the development and preclinical evaluation of a vaccine candidate against ZIKV that demonstrates exceptional safety and induces rapid and robust immunity. Our chimeric virus, designated Aripo/Zika (ARPV/ZIKV), contains the precursor membrane (prM) and envelope (E) genes of ZIKV substituted for the ARPV homologs and retains the ARPV natural host restriction (i.e., replication in mosquito cells only). Vaccination with a single dose of ARPV/ZIKV elicits robust ZIKV neutralizing antibody and T-cell responses as early as one week post-vaccination. Vaccinated immune-competent and immune-compromised mice are completely protected against ZIKV-induced morbidity, mortality and in utero transmission.

## 2. Materials and Methods

### 2.1. Cell Lines and Viruses

Mosquito (C6/36) and mammalian (VERO 76, BHK-21, JEG-3, HeLa, Hs 1.Tes and HTR-8/SVneo) cells were purchased from ATCC (Manassas, VA, USA) and maintained according to ATCC guidelines. Zika virus strains PRVABC59 and DakAr D 41524 were kindly provided by Dr. Nisha Duggal (Virginia Tech, Blacksburg, VA, USA). ARPV was originally isolated from *Psorophora albipes* mosquitoes collected from the Aripo savannahs on the Caribbean Island of Trinidad [32]. The nucleic acid sequence for the novel ARPV was deposited in GenBank (accession number: MZ358890).

### 2.2. Chimera Construction and Virus Rescue

Plasmids containing ARPV NS2A-3′ untranslated region (UTR) sequences were commercially synthesized by GenScript Inc. (Piscataway, NJ, USA). The ARPV NS1-NS2A genes were cloned into a separate plasmid (vector pACYC) from products amplified from ARPV cDNA and grown in NEB Stable cells (New England Biolabs, Ipswich, MA, USA) to limit deleterious effects that ARPV sequences have on bacteria. A gBlock was commercially synthesized from IDT (Newark, New Jersey, USA) containing a partial ARPV 3′ UTR, Hepatitis Delta ribozyme (HDVr) sequence, SbfI linearization site, T7 promoter and the ARPV 5′ UTR through Capsid genes. A previously generated ZIKV infectious clone (strain PRVABC59; GenBank accession number: KU501215) [33,34] was used to amplify ZIKV prM and E genes. All DNA fragments shared a 20–25 bp complementary sequence, allowing for an efficient Gibson assembly reaction using HiFi Builder (New England Biolabs). Gibson assembly was performed for 4 h at 50 °C. The assembled product was treated with exonuclease I and lambda (λ) exonuclease to remove ssDNA and non-circular dsDNA, respectively. Rolling circle amplification (RCA) was performed with the REPLI-g RCA kit (QIAGEN, Hilden, Germany) for 6 h and 5 µg of DNA product was used to generate capped RNA using the HiScribe T7 ARCA mRNA kit (New England Biolabs). Virus was then rescued via transfection into C6/36 cells with Lipofectamine 2000 (ThermoFisher, Waltham, MA, USA) according to the manufacturer’s guidelines.

### 2.3. RNA Extraction and Reverse Transcription Quantitative PCR (RT-qPCR)

Viral RNA was extracted using QIAmp Viral RNA Mini kits (QIAGEN) according to the manufacturer’s instructions. RT-qPCR was performed using iTaq^TM^ Universal Probes One-Step kit (Bio-Rad Laboratories, Hercules, CA, USA) according to the manufacturer’s guidelines. ARPV primers ARPV-7562F (5′-CGGTGTTCATTGAGGATGAC-3′), ARPV-7714R (5′-TGATACGTCCAGGTTCGGTA-3′) and probe TR9096-P2-7680F (5′-6FAM-CGCTGCCTCATGGCAATTCG-BHQ1-3′) were used for the detection and quantification of ARPV and ARPV/ZIKV. In vitro transcribed RNA was used to generate standard curves. Primers ZIKV-1086F (5′-CCGCTGCCCAACACAAG-3′), ZIKV-1162cR (5′-CCACTAACGTTCTTTTGCAGACAT-3′) and probe ZIKV-1107-FAM (5′-6FAM-AGCCTACCTTGACAAGCAGTCAGACACTCAA-BHQ1-3′) were used for the detection and quantification of ZIKV as previously described [35].

### 2.4. Intracellualr and Extracellular Viral Replication Kinetics

Viral replication was investigated in the intracellular and extracellular fractions of infected VERO 76 cells as previously described [32].

### 2.5. Serial Passaging of ARPV/ZIKV in C6/36 Cells

Initial post-transfection rescue titers were estimated using RT-qPCR as described above. To enhance growth kinetics in C6/36 cells, ARPV/ZIKV was serially passaged 14 times in triplicate in C6/36 cells by infecting 100 µL of virus harvested from the previous passage replicate into a new 80% confluent 25 cm^2^ culture flask and incubating for 96 h in maintenance media. Titers were quantified by RT-qPCR. A C6/36 cell passage 14 stock was generated and used for all immunization preparations.

### 2.6. Serial Passaging of Viruses in Mammalian Cells

ARPV, ARPV/ZIKV and ZIKV (strain DakAr D 41524) were each serially blind-passaged five times in VERO 76 and BHK-21 cells in triplicate to confirm retention of the desired host restriction phenotype. For each virus stock, 100 µL of maximal dose (10^9^ GC ARPV, 10^10^ GC ARPV/ZIKV, 10^5^ GC ZIKV) was inoculated onto a 90% confluent 25 cm^2^ culture flask in triplicate and incubated for 1 h. For passage 1, the inoculum was not removed and maintenance medium was applied to each flask before incubating for 96 h. For blind serial passages 2–5, 100 µL of the previous passage’s replicate was inoculated into a subsequent culture flask and after 1 h incubation the inoculum was removed, the monolayers were washed three times with phosphate buffered saline (PBS) and maintenance medium was added before incubating for 96 h. Given the stability of ARPV and ARPV/ZIKV in culture media and their vertebrate cell entry phenotype, inocula were removed from passages 2–5 to limit the detection of residual, carryover inoculum virus. Viral titers were quantified from each passage using RT-qPCR as described above.

### 2.7. SDS-PAGE and Western Blot

C6/36 or VERO 76 cells were infected with either ARPV, ZIKV (strain PRVABC59), ARPV/ZIKV, or inoculated with culture medium as a negative control and incubated for 72 h. Cells were lysed using cold RIPA buffer with 1X Halt^TM^ Protease Inhibitor Cocktail (Thermo Fisher Scientific) and supernatants were collected. A Bradford assay was performed using Coomassie Protein Assay Reagent (Thermo Fisher Scientific, Waltham, MA, USA) to quantify total protein. Protein samples were mixed with 4X Laemmli sample buffer (200 mM Tris-HCl (pH, 6.8), 9% SDS, 20% β-mercaptoethanol, 40% glycerol and 0.2% Bromophenol Blue) and denatured at 100 °C for 5 min. Ten µg of total protein per well was loaded into 12% SDS-PAGE gels (Bio-Rad Laboratories) and separated at 130 V. Proteins were then transferred onto a nitrocellulose membrane using a Mini Trans-Blot electrophoretic cell (Bio-Rad Laboratories) and blocked with 5% non-fat milk in TBST (20 mM Tris (pH, 7.4), 150 mM NaCl and 0.05% Tween 20) for 1 h. The membrane was probed with either rabbit anti-beta(β)-Actin antibody diluted 1:5000, or rabbit anti-ZIKV E antibody diluted 1:1000 for 1 h, followed by HRP-conjugated goat anti-rabbit IgG antibody for 40 min (all antibodies from GeneTex Inc., Irvine, CA, USA). Membranes were developed using the *femto*LUCENT^TM^ PLUS-HRP Kit (G-Biosciences, St. Louis, MO, USA) and exposed to autoradiography film to detect chemiluminescent signals.

### 2.8. Measuring ZIKV E Protein Expression by Flow Cytometry

Mosquito (C6/36) and mammalian (VERO 76, BHK-21, JEG-3, HeLa, Hs 1.Tes, HTR-8/SVneo) cells were inoculated with ARPV, ARPV/ZIKV, ZIKV DakAr D 41524, or PBS as a negative control. Cultures were incubated for 2–3 days post-infection (dpi). Once 50% of a cell type exhibited cytopathic effects (CPE), cells were trypsinized and made into single cell suspensions before being fixed with 4% paraformaldehyde and permeabilized with 0.1% Tween 20. Cells were stained with anti-ZIKV E protein antibody according to the manufacturer’s recommendations, followed by a secondary antibody conjugated with APC (Sino Biological, Beijing, China). An ImageStream flow cytometer was used to measure fluorescence signals for 10,000 cells, as well as simultaneously imaging each cell that was sampled.

### 2.9. Animal Experiments

All experiments were performed according to Virginia Tech approved IACUC protocols. ARPV, ARPV/ZIKV, or ZIKV inoculums used in animal experiments were prepared from infected C6/36 cell cultures. ARPV/ZIKV inoculums were concentrated from ten 150 cm^2^ flasks as previously described [29]. Inoculums were diluted with PBS to achieve the desired dosage and titers were assessed after inoculation by plaque assay on VERO 76 cells or RT-qPCR as described above.

### 2.10. Neurovirulence in Suckling Mice

Between four- and five-day-old CD-1 suckling mice (n = 12 to 18) (Charles River Laboratories, Wilmington, MA, USA) were inoculated by intracranial (i.c.) inoculation with maximal doses of ~10^9^ genome copies (GC) of ARPV/ZIKV or ARPV, or with 1.5 × 10^4^ PFU ZIKV (strain DakAr D 41524), or PBS. Following inoculation, mice were weighed daily as a group and monitored for signs of disease. At 3 dpi, 7 dpi and 14 dpi, brain samples were collected in triplicate from each group (n = 3) for virus quantification and histopathologic evaluation. Mice were euthanized when they exhibited any of the criteria for a humane endpoint (poorly responsive, extreme lethargy, seizures, or paralysis).

### 2.11. Histology

Brain samples were formalin-fixed and sectioned at three points, including rostrally at the origin of the lateral ventricles, midway at the hippocampus and thalamus and caudally at the cerebellum/brainstem. Tissues were processed and paraffin-embedded by the ViTALS diagnostic lab (Virginia Tech, Blacksburg, VA, USA). Slides were blinded, stained with H&E and scored by a board-certified veterinary pathologist. All sections were graded individually from 0 to 3 for amount of inflammation, necrosis and/or evidence of demyelination. A score of 0 was equal to no lesions, 1 to mild lesions, 2 to moderate lesions and 3 to severe lesions. A composite score was then generated for each section of brain by summing the individual scores for inflammation, necrosis and demyelination.

### 2.12. Small Animal Hemogram

Six-week-old C57BL/6 mice (n = 6) (Jackson Laboratory, Bar Harbor, ME, USA) were inoculated subcutaneously (s.c.) with 10^10^ GC ARPV, 10^11^ GC ARPV/ZIKV, 10^5^ PFU ZIKV DakAr D 41524, or PBS. Mice were euthanized from each group 7 dpi and bled by cardiac puncture. Blood samples were collected in BD Microtainer^TM^ K_2_ EDTA tubes (BD Biosciences, San Jose, CA, USA) for small animal hemogram analysis by the ViTALS diagnostic lab (Virginia Tech). Table S1 shows the 17 blood components measured.

### 2.13. Vaccine Efficacy Studies

Immunogenicity and protective efficacy of ARPV/ZIKV was evaluated in immune-competent C57BL/6 and immune-compromised IFN-αβR^−/−^ mice (Jackson Laboratory, Bar Harbor, ME, USA). Four-week-old mice were randomly divided into 6 groups of n = 6, except for the C57BL/6 ARPV/ZIKV inoculum group, where n = 12. Mice were vaccinated s.c. with maximal doses of either 10^12^ GC ARPV, 10^12^ GC ARPV/ZIKV, 10^8^ GC of ZIKV PRVABC59, or PBS. Mice were monitored daily for weight loss and signs of disease for two weeks post-immunization. Blood was collected retro-orbitally from the venous sinus at 7 and 28 days post-vaccination (dpv) and serum was stored for titration of ZIKV-specific neutralizing antibodies by the plaque reduction neutralization test (PRNT).

Mice were challenged 30 dpv with a lethal dose of 10^5^ PFU (10^6^ GC) ZIKV DakAr D 41524 administered subcutaneously. DakAr D 41524 was used as the challenge strain because of its low passage history and retention of its murine pathogenicity, including lethality. In each study, one group of mice (n = 6) initially immunized with PBS was challenged with the lethal ZIKV dose to serve as an unvaccinated control (sham-vaccinated group) and one group of mice (n = 6) initially immunized with PBS was administered PBS rather than ZIKV at challenge, in order to serve as healthy controls (PBS-vaccinated unchallenged group). To evaluate efficacy in C57BL/6 mice, we employed a regimen of anti-mouse IFNAR-1 monoclonal antibody clone MAR1-5A3 (Leinco Technologies, Fenton, MO, USA) to transiently inhibit Type I IFN (IFN-α/β) prior to ZIKV challenge. A dose of 2.5 mg antibody was administered intraperitoneally to each mouse one day prior to challenge and a dose of 1 mg per mouse was administered 1 and 4 days post-challenge (dpc).

Mice were monitored daily for weight loss and signs of disease for two weeks post-challenge. Blood was collected retro-orbitally from the venous sinus of alternating mice in each group for 1–4 dpc in C57BL/6 mice, or on 1 dpc and 3 dpc in IFN-αβR^−/−^ mice, for viremia quantification. Mice were euthanized when poorly responsive or exhibited severe lethargy, paralysis, or weight loss greater than 20% of their original 0 dpc body weight. Surviving mice were euthanized 14 dpc and bled by cardiac puncture. Sera from these mice was processed for ZIKV-specific neutralizing antibody (nAb) titers by PRNT.

### 2.14. Protective Efficacy of ARPV/ZIKV against In Utero ZIKV Transmission

Four-week-old IFN-αβR^−/−^ mice were inoculated with PBS to serve as unvaccinated unchallenged controls (healthy control mice; n = 8) or unvaccinated challenged controls (sham-vaccinated group; n = 10), or vaccinated s.c. with ARPV/ZIKV (n = 10) as described above. Dams were bled 29 dpv to measure ZIKV-specific nAb titers via PRNT. Mice were mated 30 days post-immunization and monitored daily for vaginal plugs. Mouse embryonic development was started upon plug detection (E0.5). Mice were challenged s.c. with 10^5^ PFU of Dakar D 41524 at E10.5. Dams were weighed daily for six dpc and bled at two dpc (E12.5) to quantify viremia. Dams were sacrificed six dpc (E16.5). Neonatal weights were measured immediately. Maternal brain, spleen and placental tissues were collected. Neonatal brains were collected via decapitation. Harvested tissues were homogenized in 1 mL of Dulbecco’s modified Eagle medium (DMEM) using a TissueLyser II (QIAGEN) for 5 min at 28 Hz and clarified by centrifugation at 753× *g* for 10 min. Virus titers were measured in blood sera and tissue homogenate supernatants by plaque assay.

### 2.15. T-Cell-Mediated Responses to ARPV/ZIKV

Four-week-old C57BL/6 mice were randomly divided into 4 groups (n = 6). Mice in each group were inoculated s.c. with ARPV/ZIKV, ZIKV PRVABC59, or PBS as described above in the efficacy study. At 8 dpi, half of the mice from each group were euthanized and bled by cardiac puncture. For each sample, 400 µL of blood was mixed with 4 mL of PBS containing 5 µM EDTA and stored on ice. Spleens harvested aseptically from mice euthanized 8 dpi and 35 dpi were stored on ice in filter-sterilized PBS with 2% BSA. Spleens were prepared for in vitro analysis by intracellular cytokine staining (ICS) and immunoassay as described below. To substantiate the post-inoculation cellular response observed with splenocytes cultured in vitro, blood samples taken at 8 dpi were *stained* ex vivo by previously described methods [36] using the antibodies listed in the ICS studies below.

### 2.16. Intracellular Cytokine Staining (ICS)

Splenocytes in cRPMI were cultured in 96-well plates at approximately 6 × 10^5^ cells per well. Splenocytes cultured after 8 dpi were stimulated with 2.4 × 10^4^ PFU live ZIKV (strain PRVABC59) for 24 h or 2.2 µg of ZIKV E protein, consisting of equal parts of peptides 1, 19, 118 and 199 from peptide array NR-50553 (BEI Resources, ATCC), for 6 h. Splenocytes cultured after 35 dpi were stimulated with 2.4 × 10^4^ PFU live ZIKV (strain PRVABC59) for 24 h or PepTivator^®^ Zika Envelope Protein E1-2 (Miltenyi Biotec, Bergisch Gladbach, Germany) at a final concentration of approximately 1 µg/mL of each peptide in the mix for 6 h. BD GolgiStop^TM^ (BD Biosciences) was added during the final 5 h of stimulation to inhibit protein transport. Cells were incubated with antibodies for cell surface markers CD45, CD3, CD4, CD8 and CXCR5 (CD45 antibody from Thermo Fisher Scientific, all others from BD Biosciences). Cells were fixed and permeabilized using a Fixation/Permeabilization solution (BD Biosciences) containing 4.2% formaldehyde before intracellular staining using antibodies at a dilution of 1:200 in BD Perm/Wash™ Buffer (BD Biosciences) containing FBS and saponin. The frequencies of T_H_1 (CD4^+^ CD3^+^ T-bet^+^ IFN-γ^+^), T_H_2 (CD4^+^ CD3^+^ GATA3^+^ IL-4^+^), T_H_17 (CD4^+^ CD3^+^ RORγt^+^ IL-17^+^) and T_FH_ (CD4^+^ CD3^+^ CXCR5^+^ BCL6^+^ IL-21^+^) cells were measured by intracellular staining with panels of the relevant anti-transcription factor and anti-cytokine antibodies. Cells were analyzed by flow cytometry.

### 2.17. ProcartaPlex Immunoassay

Splenocytes harvested at 8 and 35 dpi were cultured at approximately 3 × 10^5^ cells per well and stimulated as described above. Cytokines (IFN- γ, TNF- α, IL-17A, IL-12p70, IL-4, IL-6, MCP-1 (CCL2), IL-2, IL-5, IL-10, IL-21 and RANTES (CCL5)) from cell culture supernatants were quantified using a ProcartaPlex^TM^ Multiplex Immunoassay (Thermo Fisher Scientific) according to the manufacturer’s instructions.

### 2.18. Statistical Analysis

The analyses were performed in Prism 8.2 (GraphPad). Kaplan–Meier survival curves were generated and log-rank (Mantel–Cox) tests were performed to evaluate statistical significance among groups. Data were normalized by log_10_ transformation when necessary; data distribution and variance was evaluated for normality. Data were analyzed using one-way and two-way ANOVAs where appropriate and are represented in figures as the mean ± standard deviation (SD) where relevant. Multiple comparisons were performed ad hoc using Tukey’s test. Statistical results are indicated as not significant (ns), *p* ≤ 0.033 (*), *p* ≤ 0.002 (**), *p* ≤ 0.0002 (***), and *p* ≤ 0.0001 (****).

## 3. Results

### 3.1. Chimera Characterization and In Vitro Host Restriction

**Figure 1:**
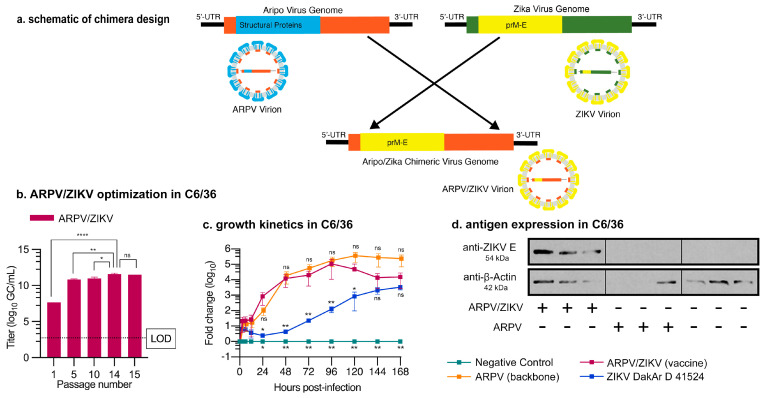
ARPV/ZIKV chimeric virus design and characterization. (**a**) Schematic of the chimeric ARPV/ZIKV vaccine genome. ARPV prM and E genes (blue) were replaced with ZIKV prM and E genes (yellow). The resultant chimeric virion expresses the structural ZIKV prM and E proteins (yellow), although the remaining ARPV genome (orange) is incompatible with replication in vertebrate cells. (**b**) Serial passaging of ARPV/ZIKV in mosquito cells (C6/36) facilitated enhanced growth for vaccine production, averaging 4.0 × 10^11^ genome copies (GC)/mL estimated by passage 14. All titers were quantified by RT-qPCR. (**c**) Growth kinetics of ARPV/ZIKV in C6/36 cells were assessed by RT-qPCR for multiple time points over 7 days. Fold change of titers normalized to the 0 h timepoint are displayed. Absolute titers are shown in Figure S1. (**d**) ARPV/ZIKV showed stable production of desired ZIKV E antigen in mosquito (C6/36) cell cultures. C6/36 cell cultures infected with various agents (absence or presence indicated by +/−) were analyzed by Western blot. Membranes were probed for ZIKV E protein or a β-actin loading control (30 s and 15 min exposure on autoradiography film, respectively). ARPV E protein showed no cross-reactivity with commercial anti-ZIKV E antibody. Linear adjustments to brightness and contrast were applied across the entire image for clarity. Radiograph images were spliced together for labelling purposes. Whole membranes are shown in Figure S2. A log_10_ transformation was applied before analysis via a one-way ANOVA and ad hoc Tukey’s test (**b**) or two-way ANOVA with Geisser–Greenhouse correction and Dunnett’s test (**c**). Error bars indicate SD of the mean. Asterisks indicate significance compared to ARPV/ZIKV: not significant (ns), *p* ≤ 0.033 (*), *p* ≤ 0.002 (**), and *p* ≤ 0.0001 (****).

**Figure 2:**
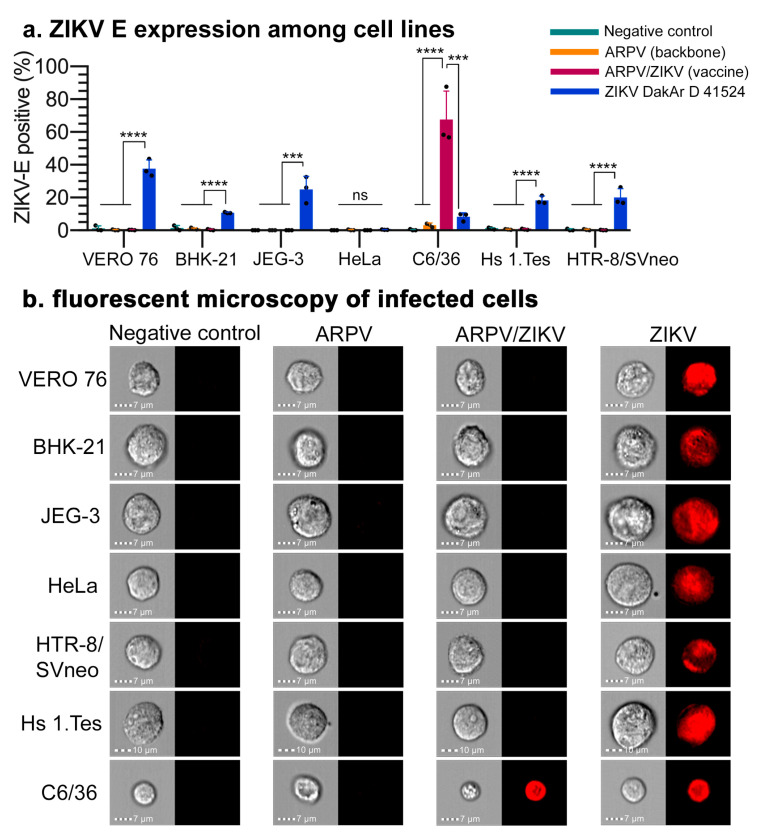
ARPV/ZIKV demonstrates a defect for replication in mammalian cells. (**a**) ARPV/ZIKV maintained its vertebrate-cell replication defect across multiple mammalian cell lines, only showing replication in mosquito cells (C6/36). Cultured cells were inoculated with one of the indicated agents or with culture media as a negative control. Viral translation within infected cells was quantified by staining with anti-ZIKV E antibody (APC-conjugated) and flow cytometric analysis. The percentage of cells positive for APC fluorescence out of the 10,000 sampled cells is indicated. Columns indicate mean values and error bars indicate SD of the mean. (**b**) Representative images of cells sampled by flow cytometry as described in (**a**). Brightfield images are left of the corresponding APC fluorescent images. Scale bars (7–10 μm) are in the bottom left of each image. Significance was determined by a one-way ANOVA. Unless otherwise marked, asterisks indicate significance compared to negative controls: not significant (ns), *p* ≤ 0.0002 (***) and *p* ≤ 0.0001 (****).

The ARPV/ZIKV chimeric virus (Figure 1a) was recovered by transfection of transcribed RNA from the cDNA clone in C6/36 cells and the initial rescue titer was estimated at 4.5 × 10^7^ GC/mL. The chimera was serially passaged fourteen times in triplicate in mosquito cells (C6/36) to optimize in vitro production of the chimeric virus and allow fixation of adaptive mutations. Passage 14 (p#14) ARPV/ZIKV showed an increase in titer to 4.0 ± 1.3 × 10^11^ GC/mL (Figure 1b) and this passage was used for subsequent experiments. ARPV/ZIKV p#14 replication kinetics were comparable to those of the backbone ARPV strain in C6/36 cells (i.e., no statistically significant differences were observed between both groups) and showed a peak titer at approximately 96 h post-infection (hpi) (Figure 1c and Figure S1). ARPV/ZIKV titers began to decline after 96 hpi whereas ARPV plateaued, but no significant differences were observed between groups. Interestingly, ARPV/ZIKV and ZIKV caused no CPE in C6/36 cells, in contrast to ARPV, which induced major CPE within three days from infection. The chimera also demonstrated stable ZIKV E protein expression in C6/36 cells when analyzed by Western blot and flow cytometry employing a ZIKV E-specific antibody (Figure 1d, Figure 2 and Figure S2, respectively).

To confirm retention of the desired host restriction of ARPV/ZIKV in mammalian cells, we evaluated ARPV/ZIKV replication by RT-qPCR and translation by flow cytometric analysis. Different mammalian cell types derived from multiple species (VERO 76, BHK-21, JEG-3, HeLa, Hs 1.Tes and HTR-8/SVneo) inoculated with ARPV or ARPV/ZIKV showed no ZIKV E protein present 48–72 h post-infection, in contrast to ZIKV-infected cells that showed robust replication and translation (Figure 2). The lack of fluorescence in ARPV-infected C6/36 cells indicates that there was no cross-reactivity between ARPV E protein and the commercial APC-conjugated ZIKV E antibody used. ARPV replication was confirmed by RT-qPCR and ARPV achieved titers of 10^10^ GC/mL under these conditions. Binding and replication studies in VERO 76 cells also showed the absence of any detectable ARPV/ZIKV replication in intracellular and extracellular fractions throughout the seven-day study period (Figure S3).

To ensure stability of the desired host restriction over multiple passages and the inability for ARPV/ZIKV to gain replication function in mammalian cells, ARPV/ZIKV was serially passaged in triplicate through mammalian (VERO 76 and BHK-21) cells five times (Figure S4a). Despite inoculating with an exceptionally high MOI of 10^4^ in passage one, no virus could be detected from passages 3–5 (Figure S4b,c); the low titers detected in passage 2 presumably reflect carryover of residual inoculum from passage 1, given the stability of this virus in culture media.

### 3.2. ARPV/ZIKV Vaccination Is Safe in Murine Models

**Figure 3:**
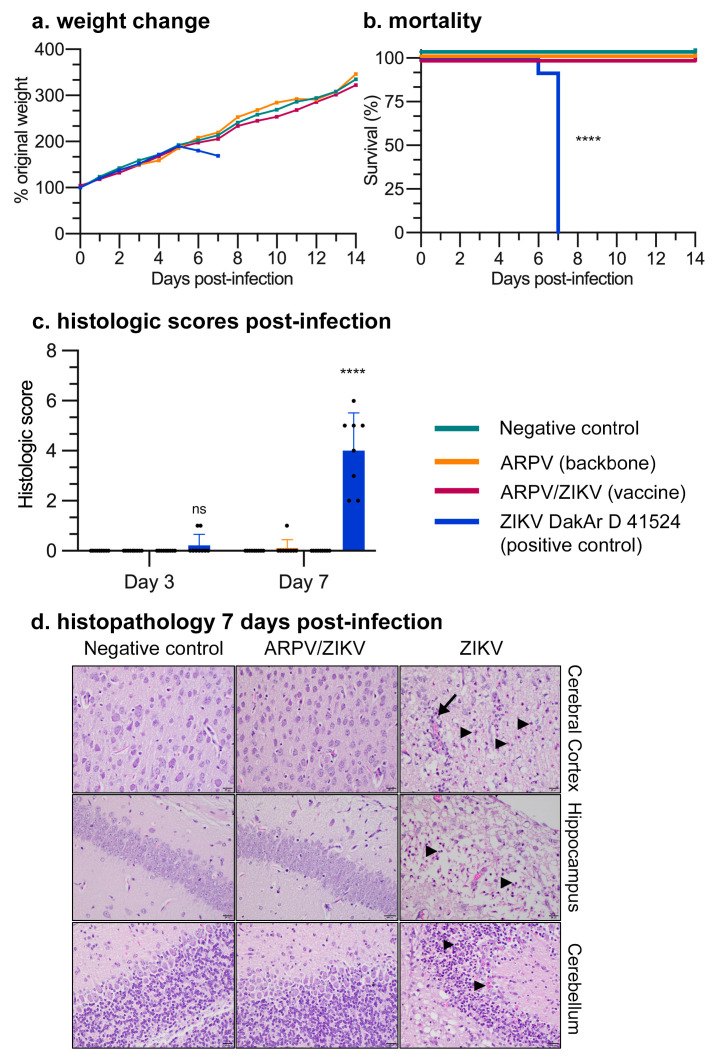
ARPV/ZIKV shows exceptional safety in a suckling mouse model. ARPV/ZIKV demonstrated no pathological effects after i.c. inoculation of between four- to five-day-old CD-1 suckling mice. Mice were inoculated with one of the indicated agents, or with saline as a negative control. (**a**) Weight change monitored over 24 days post-infection (dpi). Litters of mice were weighed as a group each day. Data points represent mean values. (**b**) Kaplan–Meier survival plot of suckling mice post-infection, with significance analyzed by log-rank (Mantel–Cox) test. No mice infected with ZIKV DakAr D 41524 survived past 7 dpi. (**c**,**d**) Mice (n = 3–7) from each group were sacrificed at 3, 7 and 14 dpi and brain samples taken for H&E staining. (**c**) Sections were graded according to amount of inflammation (0–3), necrosis (0–3) and evidence of demyelination (0–3). Higher composite scores reflect more severe histologic lesions. Columns indicate mean values and error bars indicate SD of the mean. Circles represent individual mouse data points. Significance was determined by two-way ANOVA. (**d**) Representative images (40× magnification) taken 7 dpi. The cerebral cortex is highlighted in the top panels, hippocampus in the middle panels and cerebellum in the bottom panels. Indicated are regions of neuronal necrosis (arrowheads) and inflammation (arrow). Unless otherwise marked, asterisks indicate significance compared to saline-inoculated negative controls: not significant (ns), *p* ≤ 0.0001 (****).

**Figure 4:**
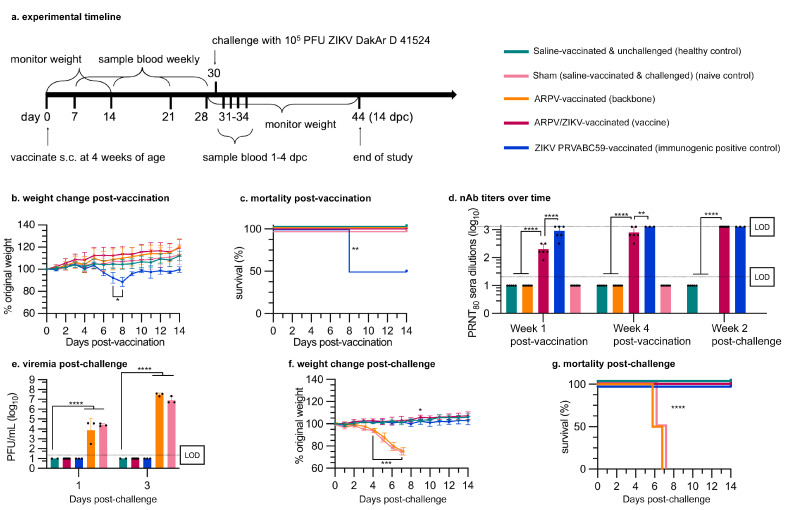
Single-dose ARPV/ZIKV vaccination was safe and provided complete protection against lethal ZIKV challenge in IFN-αβR^−/−^ mice. Four-week old IFN-αβR^−/−^ mice (n = 6) were immunized s.c. at day 0 post-vaccination. Vaccination with mouse-attenuated ZIKV PRVABC59 served as an immunogenic positive control. Mice were administered a lethal WT ZIKV challenge 30 days later, except for healthy control mice which were injected with saline at challenge. (**a**) Experimental design. (**b**) Weight change of mice for 14 days post-vaccination (dpv). ARPV/ZIKV-vaccinated mice did not differ significantly from healthy controls. ZIKV-vaccinated mice lost significant weight compared to healthy controls by *p* ≤ 0.033 at 7–8 dpv. (**c**) Kaplan–Meier survival plot of mice post-vaccination. (**d**) ZIKV-specific nAb titers measured by PRNT 7 dpv, 28 dpv and 14 days post-challenge (dpc). Values represent the reciprocal of the highest serum dilution able to reduce ZIKV plaque formation by 80%. Dotted lines represent the 20-fold or 1280-fold dilution limits of detection (LOD). (**e**–**g**) ARPV/ZIKV-vaccinated mice were completely protected from morbidity and mortality post-challenge. (**e**) Viremia at 1 and 3 dpc (early and peak viremia) was quantified by plaque assay. LOD is 100 PFU/mL. (**f**) Weight change of mice post-challenge. ARPV- and sham-vaccinated mice differed from healthy controls by *p* ≤ 0.0002 from 3–7 dpc. ARPV/ZIKV-vaccinated mice only differed significantly from healthy controls at 9 dpc, where ARPV/ZIKV-vaccinated mice gained slightly more weight than control mice. (**g**) Kaplan–Meier survival plot of mice post-challenge. (**b**,**f**) Data points indicate mean values. Error bars indicate SD of the mean. (**d**,**e**) Columns indicate mean values and error bars indicate SD of the mean. Significance was determined by log-rank (Mantel–Cox) test (**c**,**g**), one-way ANOVA (**b**,**d**,**f**), or two-way ANOVA (**e**). Unless otherwise marked, asterisks indicate significance compared to healthy controls: not significant (ns), *p* ≤ 0.033 (*), *p* ≤ 0.002 (**), *p* ≤ 0.0002 (***) and *p* ≤ 0.0001 (****).

The exceptional safety of ARPV/ZIKV vaccination was demonstrated in vivo by i.c. inoculation of suckling CD-1 mice (Figure 3) and s.c. inoculation of immune-competent (i.e., C57BL/6) (data not shown) and immune-compromised (i.e., IFN-αβR^−/−^) (Figure 4a–c) mice. Litters of between four- and five-day-old CD-1 suckling mice were injected i.c. with ~10^9^ GC of ARPV and ARPV/ZIKV, ZIKV (1.5 × 10^4^ PFU) as a positive control, or saline as a negative control. ARPV and ARPV/ZIKV inoculated suckling mice showed no signs of disease and gained weight at a comparable rate to mice inoculated with saline (Figure 3a). Pups inoculated with ZIKV (strain DakAr D 41524) gained weight at a relatively normal rate for five dpi (Figure 3a), before experiencing weight loss alongside rapid progression of clinical symptoms until day 7, when a humane endpoint was reached for euthanasia (Figure 3a,b). At 3, 7 and 14 dpi, representative brain samples (n = 3) were collected from each group (with the exception of ZIKV-infected mice, which succumbed to illness before 14 dpi). Brains were sectioned to include a frontal section to capture the periventricular stem cells, a slightly more caudal section to include hippocampus and thalamus and a very caudal section to include cerebellum and brainstem, before H&E staining. The saline-, ARPV- and ARPV/ZIKV-inoculated suckling mice exhibited no significant histologic abnormalities, in contrast to ZIKV DakAr D 41524-exposed mice. The highest composite scores (reflecting more severe histologic lesions) were reached at 7 dpi in ZIKV-infected mice (Figure 3c). Images taken from the representative groups at low magnification (2×, data not shown) showed that, by 7 dpi, ZIKV-infected brains exhibited severe lesions presenting with multifocal and often severe loss of cerebrocortical distinction, as well as loss of hippocampal structure. At 40× magnification (Figure 3d), sections showed severe neuronal necrosis with mild and often perivascular inflammation. In ZIKV-infected brains, foci of vacuolation suggestive of demyelination were observed occasionally, with such observations more prominent in sections of the cerebellum.

ARPV/ZIKV also demonstrated a high degree of safety in four-week-old immune-competent and -compromised mice. Four-week-old IFN-αβR^−/−^ mice (n = 6) (Figure 4a–c) and C57BL/6 mice (n = 12) (data not shown) were evaluated for weight loss and survival following a single subcutaneous immunization with ARPV, ARPV/ZIKV, ZIKV PRVABC59, or saline. Given that no licensed ZIKV vaccine is currently available, a mouse-attenuated ZIKV PRVABC59 was instead used as an immunogenic positive control, since this strain can induce a robust immune response without causing mortality in adult IFN-αβR^−/−^ mice [37]. In C57BL/6 mice, there were no significant differences in weight change or survival among immunization groups post-vaccination compared to saline-vaccinated unchallenged controls. However, IFN-αβR^−/−^ mice immunized with mouse-attenuated ZIKV PRVABC59 experienced 50% mortality (Figure 4c), despite this virus’ attenuation in mice [37]. No morbidity nor mortality was observed after ARPV/ZIKV vaccination of IFN-αβR^−/−^ mice. ARPV/ZIKV-vaccinated mice showed no significant differences in weight change or survival compared to saline-vaccinated controls (Figure 4b,c).

Hematological effects of ARPV/ZIKV immunization on 4-week-old C57BL/6 mice were assessed by means of a complete blood count (CBC) analysis performed on blood samples collected 7 dpi with either saline, ARPV, ARPV/ZIKV, or ZIKV DakAr D 41524. As shown in Table S1, there were no significant differences in the majority of blood parameters amongst the treatment groups, suggesting that systemic inflammation and/or bone marrow effects are not significant contributors to ZIKV pathogenesis in this model. Moreover, the administration of the vaccine was not associated with significant systemic inflammation or bone marrow effects.

### 3.3. APRV/ZIKV Protects C57BL/6 and IFN-αβR^−/−^ Mice against ZIKV-Induced Disease

**Figure 5:**
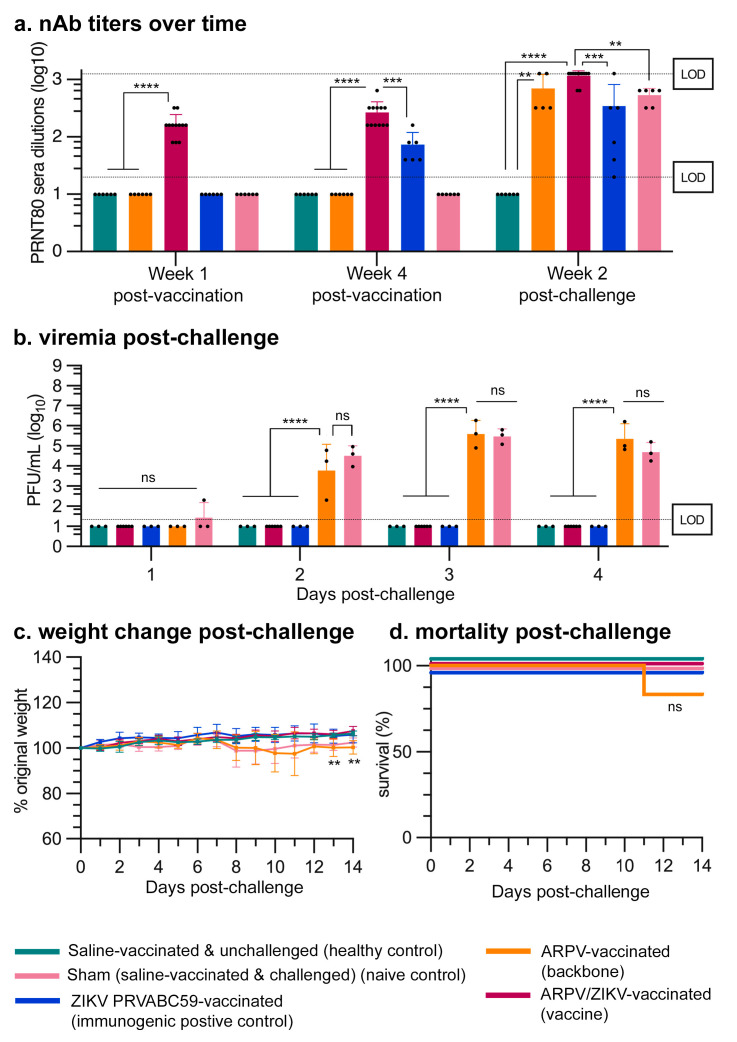
Single-dose ARPV/ZIKV vaccination provided complete protection against ZIKV challenge in C57BL/6 mice. Four-week old C57BL/6 mice (n = 12 for ARPV/ZIKV; n = 6 for all other groups) were immunized at day 0 post-vaccination. Vaccination with mouse-attenuated ZIKV PRVABC59 served as an immunogenic positive control. Mice were administered a WT ZIKV challenge 30 days post-vaccination (dpv), except for healthy control mice, which were injected with saline at challenge. Type I IFN (IFN-α/β) was transiently inhibited using a MAR1-5A3 in vivo antibody blockade prior to ZIKV challenge. (**a**) ZIKV-specific nAb titers measured by PRNT 7 days post-vaccination (dpv), 28 dpv and 14 days post-challenge (dpc). Values represent the reciprocal of the highest serum dilution able to reduce ZIKV plaque formation by 80%. Dotted lines represent the 20-fold or 1280-fold dilution limits of detection (LOD). (**b**–**d**) ARPV/ZIKV-vaccinated mice were completely protected from morbidity and mortality post-challenge. (**b**) Viremia was measured 1–4 dpc by plaque assay. LOD is 100 PFU/mL. (**c**) Weight change of mice post-challenge. ARPV-vaccinated mice differed from healthy controls on 13–14 dpc by *p* ≤ 0.002. (**d**) Kaplan–Meier survival plot of mice post-challenge. (**a**,**b**) Columns indicate mean values and error bars indicate SD of the mean. Circles represent individual mouse data points. (**c**) Data points represent mean values. Error bars indicate SD of the mean. Significance was determined by one-way ANOVA (**a**,**c**), two-way ANOVA (**b**), or log-rank (Mantel–Cox) test (**d**). Unless otherwise marked, asterisks indicate significance compared to healthy controls: not significant (ns), *p* ≤ 0.002 (**), *p* ≤ 0.0002 (***) and *p* ≤ 0.0001 (****).

Four-week-old mice were immunized s.c. with a single dose of either ARPV, ARPV/ZIKV, ZIKV PRVABC59, or saline. Sera were collected 7 and 28 dpv to measure the progression of nAb titers. ARPV/ZIKV-immunized mice developed ZIKV-specific nAb titers of 200 (±98) PRNT_80_ (reciprocal of the highest serum dilution that reduced ZIKV plaque formation by 80% in vitro) in IFN-αβR^−/−^ mice (Figure 4d) and 167 (±78) in C57BL/6 mice (Figure 5a) as early as 7 dpv. By 28 dpv, ZIKV-specific nAb PRNT_80_ titers in ARPV/ZIKV-immunized mice increased to 800 (±392) in IFN-αβR^−/−^ mice (Figure 4d) and 267 (±142) in C57BL/6 mice (Figure 5a). nAb titers of ARPV/ZIKV-immunized C57BL/6 mice were higher than those of ZIKV PRVABC59-immunized control mice at all time points measured.

A single dose of ARPV/ZIKV provided complete protection from viremia (i.e., presence of infectious ZIKV particles in blood) for both IFN-αβR^−/−^ (Figure 4e) and C57BL/6 mice (Figure 5b) and from weight loss and mortality in IFN-αβR^−/−^ mice (Figure 4f,g) following ZIKV DakAr D 41524 challenge. To observe viremia in C57BL/6 mice, mice were administered an anti-mouse IFNAR-1 antibody blockade to transiently inhibit type I IFN (IFN-α/β) prior to ZIKV challenge, as described in 2.13. ZIKV PRVABC59 immunization also provided full protection from weight loss and mortality for both mouse strains post-challenge, though with the drawback of 50% mortality in IFN-αβR^−/−^ mice post-immunization. Immunization with ARPV yielded no protection from viremia, weight loss or death. At 3 dpc, sham-vaccinated IFN-αβR^−/−^ mice (mice vaccinated with saline and administered the lethal ZIKV dose at challenge) reached a peak viremia of 1.07 (±0.97) × 10^7^ plaque-forming units (PFU)/mL (Figure 4e) and sham-vaccinated C57BL/6 mice achieved a peak viremia of 3.65 (±2.57) × 10^5^ PFU/mL (Figure 5b).

### 3.4. ARPV/ZIKV Protects IFN-αβR^−/−^ Mice against In Utero ZIKV Transmission

**Figure 6:**
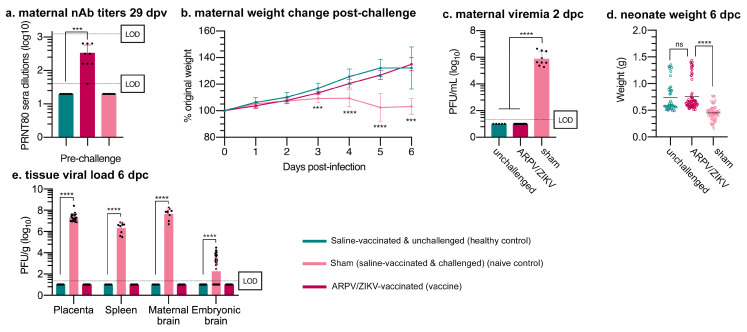
Single-dose ARPV/ZIKV vaccination of immune-compromised dams protects against in utero transmission after ZIKV challenge. Four-week-old female IFN-αβR^−/−^ mice (n = 8–10) were immunized s.c. with ARPV/ZIKV or saline 30 days prior to mating. Embryonic development was tracked upon plug detection (E0.5). (**a**) ZIKV-specific nAb titers of maternal dams was quantified by PRNT 29 days post-vaccination. Values represent the reciprocal of the highest serum dilution able to reduce ZIKV plaque formation by 80%. Dotted lines represent the lower and upper dilution limits of detection (LOD). Mice were challenged with WT ZIKV at E10.5, except healthy controls which were administered saline at challenge. (**b**) Dams were monitored daily for weight loss from 0 days post-challenge (dpc). (**c**) Maternal viremia 2 dpc (E12.5) was quantified by plaque assay on VERO 76 cells. LOD is 100 PFU/mL. Mice were euthanized 6 dpc (E16.5) to measure (**d**) neonatal weights and assess (**e**) viral load of embryonic brain and maternal placental, spleen and brain tissues by plaque assay. Columns or horizontal bars indicate mean values and error bars indicate SD of the mean. Circles represent individual mouse data points. (b) Data points represent mean values. Error bars indicate SD of the mean. Significance was determined by one-way ANOVA (**a**–**e**). Unless otherwise marked, asterisks indicate significance compared to healthy controls: not significant (ns), *p* ≤ 0.0002 (***) and *p* ≤ 0.0001 (****).

Female IFN-αβR^−/−^ mice were vaccinated with ARPV/ZIKV 30 days prior to mating. Approximately four weeks post-vaccination, these mice showed strong ZIKV-specific nAb titers (Figure 6a) and were completely protected from weight loss and viremia following challenge with a lethal dose of ZIKV DakAr D 41524 (Figure 6b,c). Neonates of ARPV/ZIKV-vaccinated dams showed no difference in mean weight compared to the neonates of healthy, unchallenged dams, but the neonates of sham-vaccinated dams weighed significantly less (Figure 6d). ARPV/ZIKV-vaccinated dams also demonstrated the complete absence of infectious ZIKV in maternal spleen, brain, or placental tissues, in contrast to mean titers of 2.20 × 10^6^, 4.82 × 10^7^ and 2.23 × 10^7^ PFU/g (respectively) estimated in sham-vaccinated controls (Figure 6e). Similarly, neonates from ARPV/ZIKV-vaccinated dams demonstrated a complete absence of ZIKV in their brains, in contrast to sham-vaccinated controls that presented viral loads of ~1.81 (±0.21) × 10^2^ PFU/g (Figure 6e).

### 3.5. ARPV/ZIKV Vaccination Generates a Robust Cell-Mediated Immune Response in Immune-Competent Mice

**Figure 7:**
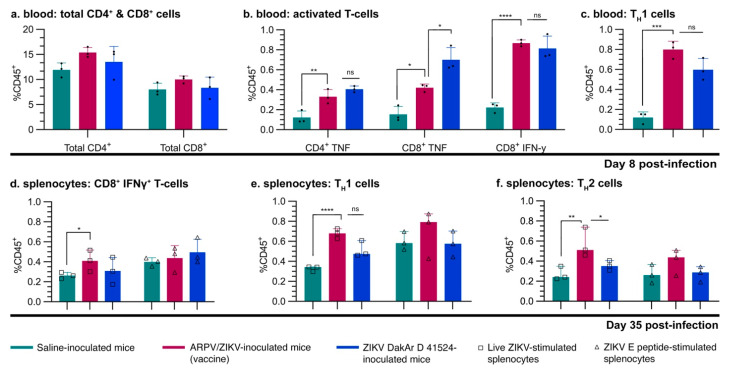
ARPV/ZIKV vaccination induces a robust T-cell-mediated immune response in immune-competent mice. Four-week-old C57BL/6 mice were inoculated s.c. with either saline, ARPV/ZIKV, or ZIKV. Splenocytes were harvested from animals (n = 3) at either 8 days post-infection (dpi) or 35 dpi and cultured in triplicate for in vitro stimulation with either live ZIKV (squares) or ZIKV E peptide mix (triangles). (**a**–**c**) Blood samples were also taken at 8 dpi and were stained ex vivo to substantiate in vitro findings. Blood samples from ARPV/ZIKV-vaccinated mice showed increases in activated CD4^+^ or CD8^+^ T cells and CD4^+^ T_H_1 subset cells. (**d**–**f**) Cultured splenocytes harvested 35 dpi and stimulated in vitro were differentiated by ICS and flow cytometry. Stimulated splenocytes derived from ARPV/ZIKV-vaccinated mice showed increases in T-cell populations of (**d**) activated CD8^+^IFNγ^+^ T cells, (**e**) CD4^+^ T_H_1 subset cells and (**f**) CD4^+^ T_H_2 subset cells. Significance was determined by one-way ANOVA (**a**–**c**) or two-way ANOVA (**d**–**f**). Columns indicate mean values and error bars indicate SD of the mean. Circles represent individual mouse data points. Unless otherwise marked, asterisks indicate significance compared to saline-inoculated negative controls: not significant (ns), *p* ≤ 0.033 (*), *p* ≤ 0.002 (**), *p* ≤ 0.0002 (***) and *p* ≤ 0.0001 (****).

**Figure 8:**
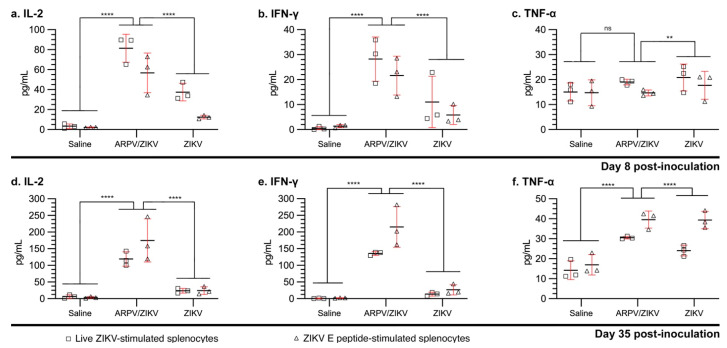
ARPV/ZIKV vaccination provides protection mediated by production of key cytokines related to antiviral response. Four-week-old C57BL/6 mice were inoculated s.c. with either saline, ARPV/ZIKV, or ZIKV. Splenocytes were harvested from animals (n = 3) at either (**a**–**c**) 8 days post-infection (dpi) or (**d**–**f**) 35 dpi and cultured in triplicate for in vitro stimulation with either live ZIKV (squares) or ZIKV E peptide mix (triangles). Cytokine levels in cell culture supernatant were determined by bead-based Luminex immunoassay. Cultured splenocytes derived from ARPV/ZIKV-vaccinated mice produced high levels of key antiviral cytokines (**a**,**d**) IL-2 and (**b**,**e**) IFN-γ at both 8 and 35 dpi and (**f**) TNF-α at 35 dpi after in vitro stimulation with ZIKV antigens. Data points represent physiological replicate values, horizontal bars indicate mean values and error bars indicate SD of the mean. Significance was determined by two-way ANOVA with ad hoc Tukey’s test. Comparisons shown were made only between the various groups stimulated with either live ZIKV, or between the various groups stimulated with ZIKV E peptide. Unless otherwise marked, asterisks indicate significance compared to saline-inoculated negative controls: not significant (ns), *p* ≤ 0.002 (**) and *p* ≤ 0.0001 (****).

Four-week-old C57BL/6 mice were inoculated s.c. with either saline, ARPV, ARPV/ZIKV, or ZIKV PRVABC59, as indicated in the efficacy studies above. At 8 dpi and 35 dpi, splenocytes were harvested and cultured for in vitro stimulation with either live ZIKV or ZIKV E peptides. T-cell populations were differentiated by ICS and flow cytometry (Figure 7). Although ARPV/ZIKV-immunized mice showed no significant increase in total CD4^+^ or CD8^+^ cells in the blood at 8 dpi (Figure 7a), they showed significantly higher proportions of activated CD4^+^ and CD8^+^ T cells (Figure 7b). Compared to saline-immunized control groups, stimulated splenocytes from ARPV/ZIKV-immunized mice expressed significantly higher proportions of ZIKV-specific T_H_1 CD4^+^ T cells at both 8 dpi and 35 dpi (Figure 7e), as well as significantly higher proportions of T_H_2 CD4^+^ T cells at 35 dpi (Figure 7f). This was confirmed at 8 dpi by ex vivo blood analysis (Figure 7c). Additionally, stimulated splenocytes derived from ARPV/ZIKV-immunized mice produced significantly more cytokines associated with viral infection than control groups at both 8 dpi and 35 dpi, with notable increases in IL-2 (Figure 8a,d), IFN-γ (Figure 8b,e) and TNF-α (Figure 8c,f). Stimulated splenocytes from ARPV/ZIKV-immunized mice also showed significant increases in IL-12p70 (8 and 35 dpi), RANTES (8 and 35 dpi), MCP-1 (CCL2) (8 and 35 dpi), IL-4 (35 dpi) and IL-6 (35 dpi), compared to saline-immunized mice (Tables S2 and S3). Preliminary studies performed with dose-matched ARPV, ZIKV and saline controls indicated that there were no or minimal statistically significant differences between ARPV and the saline-immunized controls, but significance was observed between ZIKV and saline and ZIKV and ARPV (data not shown). Taken altogether, these responses indicate a robust T-cell-mediated response to ARPV/ZIKV immunization.

## 4. Discussion

We explored a variety of in vitro and in vivo measures to characterize the safety profile of ARPV/ZIKV relative to its ARPV backbone and ZIKV. A key feature of ARPV/ZIKV is its safety due to the inherent mammalian host-restriction imposed by ARPV. Our in vitro RT-qPCR and protein expression studies demonstrated the absence of detectable ARPV/ZIKV replication in mammalian cell lines that are highly susceptible to arbovirus—specifically ZIKV—infection. Our results suggest that ARPV/ZIKV is fundamentally mammalian host-restricted, likely before the point of viral RNA amplification (Figure S3). The absence of detectable replication in mammalian cells is likely not a temperature-dependent effect, as previous studies show ARPV (i.e., vaccine backbone) was unable to replicate in mammalian cells at 29 °C [32]. Furthermore, ARPV/ZIKV was blindly serially passaged through immune-compromised mammalian cells to facilitate adaptation for replication, but there was no evidence of this gain of function for ARPV or ARPV/ZIKV, as quantified by RT-qPCR (Figure S4). Further work should be conducted to confirm the stability of the chimera’s host restriction over multiple passages in human and other vertebrate cell lines. Given the strong evidence for a complete replication defect of ARPV and ARPV/ZIKV, the likelihood of this adaptation to develop is extremely low.

ARPV/ZIKV also demonstrates exceptional safety in murine models, yielding no detectible adverse health effects after immunization. Our hemogram studies showed that administration of the ARPV/ZIKV vaccine was not associated with significant inflammation (Table S1). This study did show a significant decrease in the mean corpuscular volume of erythrocytes in circulation (microcytosis) of mice exposed to the vaccine and vector, but an even more significant decrease in those mice exposed to ZIKV. This finding is interesting, as microcytic anemia has uncommonly been reported in Zika-infected patients [38,39]. In these human cases, however, there is a concurrent anemia and thrombocytopenia, which was not identified in vaccinated and/or infected mice. Thus, the clinical significance of these findings awaits further studies to investigate whether microcytosis persists in vaccinated mice. We also examined the safety of ARPV/ZIKV in suckling mice. This model is recognized as among the most permissive environments for arbovirus replication. ARPV- and ARPV/ZIKV-inoculated mice showed no signs of illness or change in the rate of weight gain (Figure 3a,b) and no histopathological lesions were observed in brain tissues (Figure 3c,d). This is in stark contrast to ZIKV-inoculated mice, which demonstrated weight loss, death and severe neuronal necrosis and inflammation. In older mouse models, ARPV/ZIKV immunization produced no adverse events or detectable illness (Figure 4b,c). This exceptional safety, coupled with the inability for ARPV/ZIKV to replicate in mammalian cells and the stable retention of the desired ISFV host restriction in vitro, strongly supports the safety of this promising vaccine candidate.

ARPV/ZIKV immunization results in a rapid and robust immune response that provides single-dose protection from ZIKV-induced disease. Our studies show that ARPV/ZIKV is exceptionally immunogenic relative to the ZIKV-immunized controls in both immune-competent (C57BL/6) and immune-compromised (IFN-αβR^−/−^) murine models. IFN-αβR^−/−^ mice were used as a challenge model because of their increased susceptibility to disease due to their lack of a functional type I interferon response [40,41], in order to increase the rigor for evaluating vaccine efficacy. Alternatively, C57BL/6 mice were used to assess vaccine immunogenicity in an immune-competent model. In both models, strong nAb responses were observed as early as 7 dpv with ARPV/ZIKV (Figure 4d and Figure 5a), indicating that the minimum PRNT_50_ of 100 required for protection [42] was exceeded by 7 dpv. ARPV/ZIKV immunization produced higher nAb titers than all other groups, including our immunogenic positive control, at 7 and 28 dpv in C57BL/6 mice. This likely reflects, at least in part, the exceptionally high dose of ARPV/ZIKV used relative to ZIKV, which was achieved in the absence of adverse health events. In C57BL/6 mice, ZIKV NS5 fails to antagonize mouse STAT2 and so ZIKV is unable to develop viremia and induce a systemic infection [43,44]. However, ARPV/ZIKV efficacy does not depend on viral amplification within the mouse to illicit a robust immune response. Alternatively, ZIKV PRVABC59 immunization of IFN-αβR^−/−^ mice showed higher nAb responses than ARPV/ZIKV. In preliminary studies, ZIKV PRVABC59 was mouse-attenuated and generated viremia in the absence of death or disease in adult IFN-αβR^−/−^ mice; hence, it used as an immunogenic positive control in these mouse studies. The high nAb titers for ZIKV PRVABC59-immunized IFN-αβR^−/−^ mice are expected, given the enhanced susceptibility of this murine model to viral replication in the absence of an interferon type I response, which presumably allows an attenuated virus to illicit immunity similar to that following a wild-type infection. The absence of any protective effects in ARPV-immunized control mice confirm the importance of the prM and E substitutions in the ISFV backbone. Vaccines that have rapid and single-dose efficacy coupled with a high degree of safety are most desirable for the control of explosive outbreaks and for vaccination in endemic regions where frequent boosters may be financially and logistically challenging. Although ARPV/ZIKV demonstrated single-dose efficacy, further studies are needed to confirm the longevity of ARPV/ZIKV protection and determine if boosters are required to maintain long-term efficacy.

Overall, ARPV/ZIKV-immunized mice were completely protected from morbidity, in utero ZIKV transmission and mortality, after ZIKV challenge. This protection can be attributed to both robust neutralizing antibody and T-cell responses. The latter have been shown to play a critical role during ZIKV infection and vaccine-induced protection. Our studies showed both robust CD8^+^ and CD4^+^ T_H_1 cellular responses (Figure 7) with strong activation of the IL-2 and IFN-γ signaling pathways (Figure 8), a common characteristic of viral vectored vaccines [11,12,15,45]. Further work is required to quantitatively examine the T-cell response to ARPV/ZIKV vaccination and dissect its role in mediating protection. Although our approach assessed cellular responses to ZIKV via stimulation of immune splenocytes with ZIKV peptides or virions, performing a stimulation control with ARPV would be useful to assess the full magnitude of the T-cell response to ARPV/ZIKV immunization.

Additionally, a key factor underlying vaccine availability is the ability to manufacture adequate numbers of doses quickly. An advantage of ARPV/ZIKV is its ease of production and high titers achieved in cell culture at low biosafety containment. Although these cell culture-based systems are readily available for commercial production and purification of the required efficacious dose, the mosquito cells required for ARPV/ZIKV production are not FDA-approved substrates and this presents a unique challenge for the approval of ISFV chimeric vaccines.

In summary, optimizing vaccine availability through improved production, stability, safety, immunogenicity, delivery and efficacy is critical to our success in the defense against pathogen emergence. As such, we employed a novel ISFV vector and demonstrated the exceptional safety and single-dose efficacy of our chimeric vaccine, ARPV/ZIKV, in multiple murine models. The development of additional ISFV platforms further supports the viability of ISFV chimeric platforms as a commercially useful option and reduces concerns regarding anti-vector immunity if these platforms are licensed. This study also reports several significant advances characterizing the safety, immunogenicity and efficacy of insect-specific flavivirus chimeric vaccines, including (i) important quantitative in vitro and in vivo safety studies, (ii) cellular responses to vaccination with this chimera and (iii) T-cell-mediated cytokine and chemokine responses in immune cells. Our efficacy data are also strongly supported by and expand upon, recent studies that explored the Binjari virus as an ISFV vaccine vector [25,26,27,46]. Although both platforms are similar, small phenotypic differences exist, including the peak titers achieved in cell culture, the nAb titers achieved after immunization, or the fact that some Binjari virus chimeras have been shown to require boosters or adjuvants to achieve adequate protection [25,26,27,46]. Binjari virus chimeras have been developed for several flaviviruses and it is not unlikely that ARPV chimeras with other flaviviruses will require comparable modifications to be effective. Further studies are needed to adequately compare both ISFV platforms. Lastly, further studies are warranted to thoroughly assess ARPV/ZIKV’s potential as a vaccine candidate. Long-term protection studies are needed to evaluate the longevity of the antibody response and efficacy. Additionally, although murine models of ZIKV pathogenesis remain reliable and reproducible, NHP studies are required to evaluate this vaccine candidate given their greater physiological and immunological similarity to humans.

## Data Availability

All virus and vaccine strains are available from the authors upon request.

## Supplementary Materials

The following are available online at https://www.mdpi.com/article/10.3390/vaccines9101142/s1, Figure S1: ARPV growth kinetics in C6/36 cells, Figure S2: Full Western blot membranes corresponding to Figure 1d, Figure S3: ARPV/ZIKV is likely host restricted in mammalian cells prior to viral genome RNA amplification, Figure S4: ARPV/ZIKV retains its replication defect over multiple passages in mammalian cells, Table S1: Administration of the ARPV/ZIKV vaccine is not associated with significant systemic inflammation, Table S2: Splenocytes stimulated with live Zika virus produce significant quantities of key cytokines related to antiviral response, Table S3: Splenocytes stimulated with Zika virus peptides produce significant quantities of key cytokines related to antiviral response.
