# Supplementary material for: Enemy of My Enemy: A Novel Insect-Specific Flavivirus Offers a Promising Platform for a Zika Virus Vaccine
Source: Vaccines (Basel). 2021 Oct 7;9(10):1142. doi: 10.3390/vaccines9101142 (PMC8539214; doi:10.3390/vaccines9101142)
Supplement: Supplementary file 1 [file vaccines-09-01142-s001.zip › vaccines-1375042-supplementary.pdf]

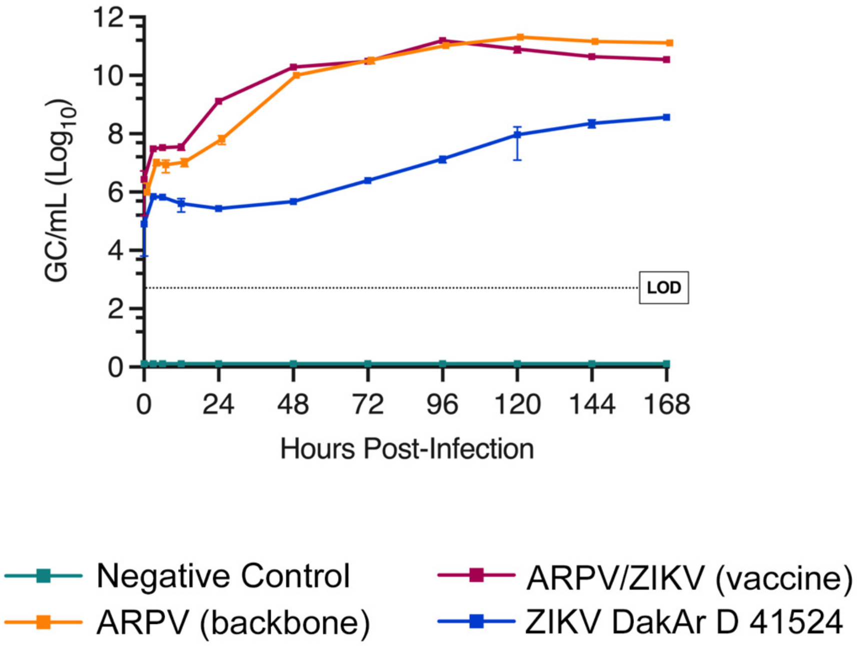

Supplemental Figure S1. ARPV/ZIKV growth kinetics in C6/36 cells. Growth kinetics of ARPV, ARPV/ZIKV, and ZIKV in C6/36 cells were assessed by RT-qPCR at multiple time points over 7 days. ARPV/ZIKV kinetics and titers achieved were comparable to the ARPV backbone virus. Shown above are the absolute titers of the same viral supernatant samples used to construct the fold change graph displayed in Figure 1c. Error bars indicate SD of the mean.

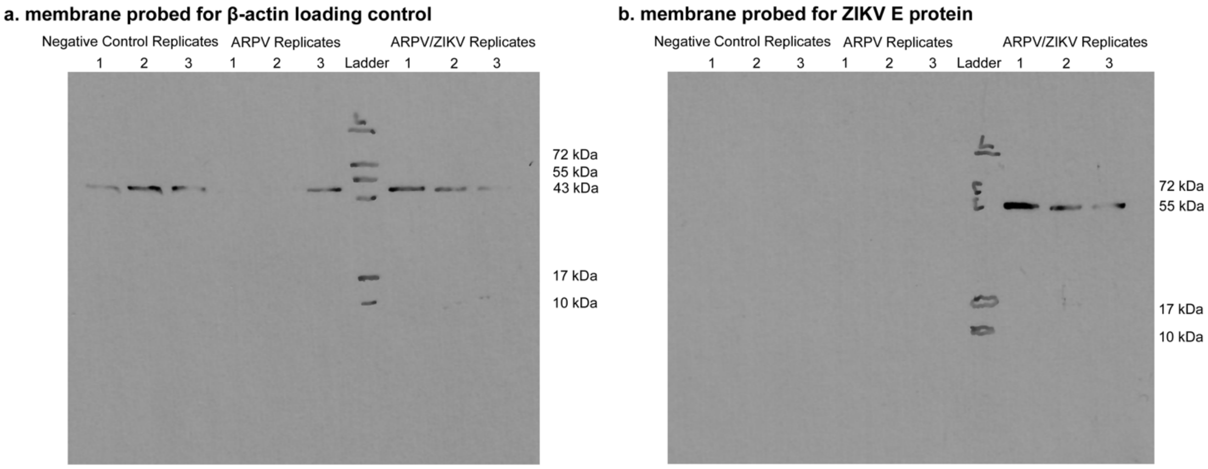

Supplemental Figure S2. Full Western blot membranes corresponding to Figure 1d. ARPV/ZIKV showed stable production of desired ZIKV E antigen in mosquito (C6/36) cell cultures. C6/36 cell cultures infected with various agents were analyzed by western blot. (**a**) Membrane was probed for a β-actin loading control (15 minute exposure on autoradiography film). (**b**) Membrane was probed for ZIKV E protein (30 s exposure on autoradiography film). ARPV E protein showed no cross-reactivity with commercial anti-ZIKV E antibody.

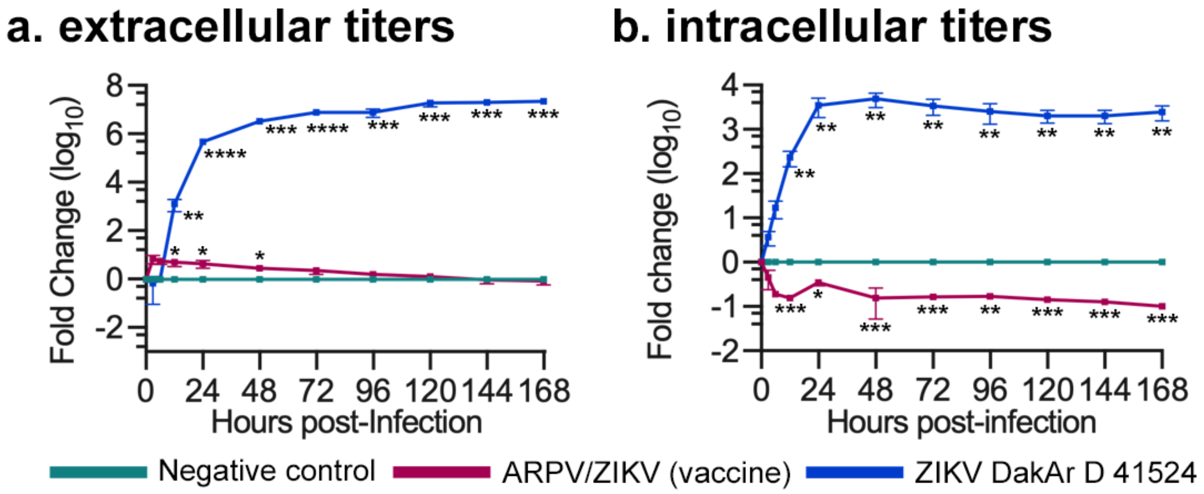

Supplemental Figure S3. ARPV/ZIKV is likely host-restricted in mammalian cells prior to viral genome RNA amplification. (**a**) Analysis of viral titers from cell culture supernatant indicated that ARPV/ZIKV produced no viable viral progeny after inoculation of VERO 76 cells. (**b**) Analysis of intracellular RNA from VERO 76 cells inoculated with ARPV/ZIKV indicated that ARPV/ZIKV is host restricted before the point of RNA amplification, as intracellular viral RNA titers failed to increase post-infection. All titers quantified by RT-qPCR. Error bars indicate SD of the mean. Significance was determined by two-way ANOVA. Unless otherwise marked, asterisks indicate significance compared to negative controls: not significant (ns), $P \leq 0.033$ (*), $P \leq 0.002$ (**), $P \leq 0.0002$ (***), $P \leq 0.0001$ (****).

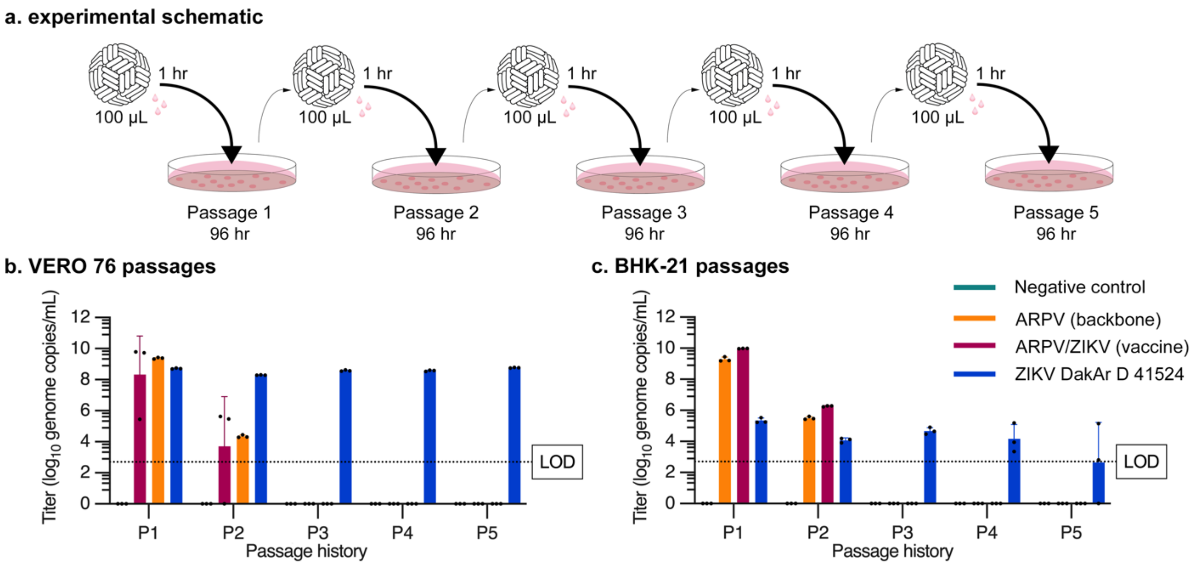

Supplemental Figure S4. ARPV/ZIKV retains its replication defect over multiple passages in mammalian cells. (**a**) Schematic of blind serial passaging of ARPV, ARPV/ZIKV, ZIKV, or supernatant from un-infected cultures (negative control). For passage 1 (P1), mammalian cells (**b**) VERO 76 or (**c**) BHK-21 were infected with virus and incubated for one hour before adding maintenance media and incubating 96 hours. For subsequent passages (P2-P5), 100 µL supernatant of the previous passage was applied to new flasks, incubated for one hour, and the monolayers washed before adding maintenance media and incubating for 96 hours. Data indicated no ability for ARPV/ZIKV to gain replication function in mammalian cells. (**b**,**c**) All viral titers were quantified by RT-qPCR. Columns indicate mean values, and error bars indicate SD of the mean. Significance was determined by one-way ANOVA and ad hoc Tukey's test. Unless otherwise marked, asterisks indicate significance compared to culture media-inoculated negative controls not significant (ns), $P \leq 0.033$ (*), $P \leq 0.002$ (**), $P \leq 0.0002$ (***), $P \leq 0.0001$ (****).

Supplemental Table S1. Administration of the ARPV/ZIKV vaccine is not associated with significant systemic inflammation.

	Saline	ARPV	ARPV/ZIKV	ZIKV DakAr D 41524
**RBC** (x10^6 cells/μL)	10.3 (0.2)	10.6 (0.3)	10.7 (0.2)	10.3 (0.3)
**Hemoglobin** (g/dL)	15.7 (0.3)	15.8 (0.4)	16.0 (0.1)	15.5 (0.5)
**Hematocrit** (%)	52.7 (1.2)	53.0 (1.3)	53.5 (0.9)	50.6 (1.9)
**MCV** (fL)	51.2 (0.5)	50.0 (0.6)*	50.0 (0.5)*	49.3 (0.8)***
**MCHC** (g/dL)	30.0 (0.3)	29.8 (0.1)	29.9 (0.4)	30.6 (0.6)
**NRBC Sysmex** (%)	0.3 (0.4)	0.7 (0.2)	0.3 (0.2)	0.4 (0.5)
**RDW-CV** (%)	16.4 (0.2)	17.0 (0.3)	17.3 (0.5)	16.7 (0.2)
**MPV** (fL)	6.3 (0.2)	6.5 (0.3)	6.2 (0.0)	6.6 (0.3)
**Reticulocyte** (%)	3.7 (0.4)	4.0 (0.9)	3.0 (0.4)	3.4 (0.4)
**Absolute Reticulocyte** (x10^3 cells/μL)	379.3 (41.9)	422.9 (83.4)	323.3 (39.6)	347.8 (45.8)
**WBC** (x10^3 cells/μL)	7.6 (0.8)	8.8 (2.4)	9.2 (0.8)	8.9 (2.3)
**Neutrophils** (x10^3 cells/μL)	0.6 (0.2)	0.7 (0.2)	0.8 (0.2)	0.8 (0.3)
**Lymphocytes** (x10^3 cells/μL)	6.8 (1.2)	7.0 (2.1)	7.6 (0.7)	7.8 (1.4)
**Monocytes** (x10^3 cells/μL)	0.5 (0.1)	0.9 (0.4)	0.7 (0.1)	0.8 (0.1)
**Eosinophils** (x10^3 cells/μL)	0.1 (0.0)	0.1 (0.1)	0.1 (0.0)	0.2 (0.2)
**Basophils** (x10^3 cells/μL)	0.0 (0.0)	0.1 (0.0)	0.1 (0.1)	0.1 (0.1)
**Platelets** (x10^3 cells/μL)	1031.2 (53.3)	932.5 (196.2)	1312.3 (102.5)	909.0 (276.7)

[a]Data represent average values of biological replicates with standard deviation (SD). Asterisks indicate significance by one-way ANOVA with ad hoc Tukey's test: not significant (ns), P<0.033 (*), P<0.002 (**), P<0.0002 (***), P<0.0001 (****).

[b]RBC, red blood cell; MCV, mean corpuscular volume; MCHC, mean corpuscular hemoglobin concentration; NRBC, nucleated red blood cell; RDW-CV, red blood cell distribution width; MPV, mean platelet volume; WBC, white blood cell.

Supplemental Table S2. Splenocytes stimulated with live Zika virus produce significant quantities of key cytokines related to antiviral response.

Analyte	Day 8 post-infection			Day 35 post-infection		
	Saline	ARPV/ZIKV	ZIKV DakAr D 41524	Saline	ARPV/ZIKV	ZIKV DakAr D 41524
**IL-2**	3.4 (2.3)	81.5 (14.1) ****	37.5 (8.7) ****	6.3 (5.3)	119.3 (21.6) ****	23.8 (7.0) ***
**IL-4**	0.8 (0.4)	1.9 (0.5)	2.1 (1.0) *	0.8 (0.9)	3.4 (0.9) ***	2.4 (1.6) *
**IL-5**	4.2 (1.2)	3.6 (0.6)	12.7 (10.7) ****	3.9 (3.4)	8.4 (2.3)	10.2 (5.8)
**IL-6**	46.6 (5.6)	61.88 (4.8)	69.92 (20.8) **	29.5 (20.0)	63.4 (14.4) ****	50.4 (28.9) *
**IL-10**	10.8 (2.2)	13.60 (1.5)	18.0 (5.9)	19.1 (14.6)	25.1 (11.8)	25.9 (10.9)
**IL-12p70**	0.2 (0.3)	1.5 (1.0) *	1.2 (1.0)	0.4 (0.3)	3.3 (0.6) ****	1.1 (0.8)
**IL-17A**	1.1 (0.7)	0.2 (0.3)	0.5 (0.5)	1.3 (1.5)	1.6 (1.0)	1.0 (1.0)
**MCP-1**	13.5 (9.2)	24.8 (1.4) ****	22.9 (7.7) ****	13.3 (7.6)	29.9 (2.2) ****	22.4 (7.9) **
**IFN-γ**	0.5 (0.7)	28.2 (8.9) ****	11.1 (10.3) ****	0.6 (0.9)	135.6 (5.7) ****	13.6 (5.7)
**TNF-α**	15.1 (3.6)	19.0 (1.1) ****	20.8 (5.4) ****	14.2 (4.7)	30.5 (0.7) ****	24.1 (2.7) ****
**RANTES**	256 (35.6)	291.4 (2.9) **	313.5 (69.8) ****	314.5 (26.5)	343.2 (19.7) *	346.1 (39.1) *

Data represent average values (pg/mL) of biological replicates with standard deviation (SD). Asterisks indicate significance by two-way ANOVA with ad hoc Tukey's test. Significance shown is compared to saline inoculated negative controls: $P<0.033$ (*), $P<0.002$ (**), $P<0.0002$ (***), $P<0.0001$ (****).

Supplemental Table S3. Splenocytes stimulated with Zika virus peptides produce significant quantities of key cytokines related to antiviral response.

Analyte	Day 8 post-infection			Day 35 post-infection		
	Saline	ARPV/ZIKV	ZIKV DakAr D 41524	Saline	ARPV/ZIKV	ZIKV DakAr D 41524
**IL-2**	2.1 (0.4)	56.7 (19.8) ****	12.4 (1.8) **	3.7 (2.6)	174.9 (64.8) ****	24.3 (11.4)
**IL-4**	1.8 (0.5)	1.9 (0.4)	1.6 (0.8)	1.9 (1.3)	2.8 (0.7)	2.5 (1.4)
**IL-5**	4.6 (1.5)	2.4 (0.5)	3.9 (1.9)	3.9 (2.4)	5.2 (1.0)	4.8 (1.9)
**IL-6**	30.6 (6.1)	21.9 (6.6)	21.8 (3.9)	19.3 (8.9)	55.7 (10.9) ***	27.1 (20.6)
**IL-10**	1.5 (1.8)	0.1 (0.1)	0.7 (0.6)	5.5 (4.8)	10.2 (7.7)	3.3 (5.0)
**IL-12p70**	0.9 (0.2)	1.2 (0.0)	0.8 (0.2)	1.2 (0.2)	1.9 (0.3)	1.1 (0.3)
**IL-17A**	1.4 (0.4)	0.7 (0.2)	0.7 (0.2)	1.2 (0.7)	1.4 (0.4)	0.8 (0.3)
**IL-21**	3.7 (0.0)	3.7 (0.0)	3.7 (0.0)	3.7 (0.1)	3.7 (0.0)	3.6 (0.0)
**MCP-1**	14.9 (5.4)	11.8 (1.4)	10.5 (3.3)	14.5 (1.7)	28.1 (1.8) ****	25.2 (11.2) ****
**IFN-γ**	1.4 (0.6)	21.6 (7.8) ****	5.8 (3.8) **	1.9 (0.8)	215.4 (60.9) ****	26.7 (15.2) ***
**TNF-α**	14.7 (5.1)	14.7 (1.2)	17.7 (5.6) **	17.0 (5.1)	39.6 (4.3) ****	39.4 (4.3) ****
**RANTES**	325.2 (67.2)	337.7 (40.3)	333.7 (71.6)	458.0 (24.6)	1550.3 (1689.9)	571.8 (111.1)

Data represent average values (pg/mL) of biological replicates with standard deviation (SD). Asterisks indicate significance by two-way ANOVA with ad hoc Tukey's test. Significance shown is compared to saline inoculated negative controls: $P<0.033$ (*), $P<0.002$ (**), $P<0.0002$ (***), $P<0.0001$ (****).
